# Ammonol in Malarial Paroxysm

**Published:** 1895-11

**Authors:** Cyrus Edson

**Affiliations:** New York, President of Board of Pharmacy, City of New York; Member New York Academy of Medicine, Member New York County Medical Society, Past Health Commissioner of New York State and New York City, etc.


### Selections and Abstracts.

### AMMONOL IN MALARIAL PAROXYSM.

By CYRUS EDSON, M. D., New York,

President of Board of Pharmacy, City of New York; Member New York Academy of Medicine, Member New York County Medical Society, Past Health Commissioner of New York State and New York City, etc.

Many writers have called attention to the unfavorable effects of quinia when administered during the paroxysm of malarial fever. The drug then seems to increase the headache and to have a rather bad effect on the general condition of the patient.

It has been effectually demonstrated that the proper time for quinine is during the period of intermission or remission, and no one can deny that quinine is a *sine qua non* in the treatment of malaria, when thus used. The treatment of the malarial paroxysm, *i.e.*, the algid and febrile stage of the disease, has not received the attention it deserves at the hands of medical men. Usually, the patient is packed in heavy blankets and permitted to shake, burn and sweat in succession, until the paroxysm wears itself out. Not only is this discomfort unnecessary, but the physician, by permitting it, loses valuable time and makes subsequent treatment more difficult. Careful, judicious treatment of the paroxysmal stage of malarial fevers will relieve much suffering and very materially aid subsequent treatment.

I propose to outline in detail the treatment of a typical case of intermittent fever, and I will preface my description with the statement that I have followed this treatment in a large number of cases, obtaining such invariably successful results as to lead me to adopt it as a regular routine practice in all cases of malarial fever.

The treatment is commenced, during the first chill, if possible, by administration of from five to fifteen grains of ammonol, the dose depending on the age of the patient, a child of ten years being given the minimum dose, and one grain being added for each subsequent year for older persons up to the maximum dose of fifteen grains.

The remedy is given in powder form, dry on the tongue, and washed down with a hot toddy of whisky, rum or gin, sweetened to the taste, and not very strong. The amount of the alcoholic liquor should vary from a teaspoonful to two tablespoonfuls, in about four or five times the amount of hot water. This quantity should be determined by the age of the patient. The good effect of this dose should be apparent within an hour. The headache disappears, as if by magic; in many cases, the chill is shortened and the fever and sweat completely aborted. At the expiration of an hour from the time of the administration of the first dose, a second dose of the same quantity of ammonol, followed by half the amount of toddy, should be given. Occasionally, the second dose may consist of half the quantity of ammonol first given; this if the patient seems so completely relieved as not to require medicine,

My first action, after a cessation of the paroxysmal stage. is to exhibit a dose of calomel rubbed up with soda bi-carb., and the following prescription indicates doseage:

℞.
Hydrarg. chloridi mite.,
Sodæ bi-carb., aa grs. iii. to vj.
M. Sig.—One dose.

In very young children gray powder in dose of three-quarter grains may be best substituted for the calomel. During the stage of intermission I give a dose of six to twelve grains of quinine, preferably in liquid form, *i. e.*, dissolve by aid of sulphuric acid in water, as follows:

℞.
Quinia sulph., gr. xij.
Acid sulph., dil. qs., ft. sol.
Aquæ, ℥i.
M. Sig.—One dose; to be taken after cessation of fever.

In a majority of cases, no secondary chill will occur until the seventh or fourteenth day, if no further treatment is given, but to clinch the driven nail, it is necessary to give two or three grains of quinine before meals, followed after eating, three times daily, with Fowler's solution of arsenic, dose, two to six drops for a week. Ten grains of ammonol should be taken at bedtime during this period, and the bowels kept open by means of a mild saline aperient, taken before breakfast in the morning.

The action of ammonol, in the malarial types of fever, seems peculiarly happy. Its good effect is perhaps largely due to the ammonia it contains. This agent has for a long time been known favorably as a malarial remedy. It unlocks the secretions, stimulates, has a selective action on the liver, and not unlikely is a destroyer of the plasmodium of malaria.

That ammonol is an effective malarial germicide can readily be proved by its action on the disease when given as the sole treatment in cases of it. I find the treatment I have given as most certain, however, and I cannot too highly recommend it. In case of remittent fever, I follow the same line of treatment, using ammonol during the stage of high fever and quinia during the remission.—*The National Board of Health Magazine.*

